# Predicting Bipolar Disorder Risk Factors in Distressed Young Adults From Patterns of Brain Activation to Reward: A Machine Learning Approach

**DOI:** 10.1016/j.bpsc.2019.04.005

**Published:** 2019-08

**Authors:** Leticia de Oliveira, Liana C.L. Portugal, Mirtes Pereira, Henry W. Chase, Michele Bertocci, Richelle Stiffler, Tsafrir Greenberg, Genna Bebko, Jeanette Lockovich, Haris Aslam, Janaina Mourao-Miranda, Mary L. Phillips

**Affiliations:** a Centre for Medical Image Computing, Department of Computer Science, London, United Kingdom; b Department of Physiology and Pharmacology, Federal Fluminense University, Niterói, Brazil; c Department of Psychiatry, Western Psychiatric Institute and Clinic, University of Pittsburgh Medical Center, University of Pittsburgh, Pittsburgh, Pennsylvania

**Keywords:** Biomarkers, Bipolar disorder, fMRI, Machine learning, Multivariate pattern, Neuroimage

## Abstract

**Background:**

The aim of this study was to apply multivariate pattern recognition to predict the severity of behavioral traits and symptoms associated with risk for bipolar spectrum disorder from patterns of whole-brain activation during reward expectancy to facilitate the identification of individual-level neural biomarkers of bipolar disorder risk.

**Methods:**

We acquired functional neuroimaging data from two independent samples of transdiagnostically recruited adults (18–25 years of age; *n =* 56, mean age 21.9 ± 2.2 years, 42 women; *n =* 36, mean age 21.2 ± 2.2 years, 24 women) during reward expectancy task performance. Pattern recognition model performance in each sample was measured using correlation and mean squared error between actual and whole-brain activation–predicted scores on behavioral traits and symptoms.

**Results:**

In the first sample, the model significantly predicted severity of a specific hypo/mania-related symptom, heightened energy, measured by the energy manic subdomain of the Mood Spectrum Structured Interviews (*r =* .42, *p =* .001; mean squared error = 9.93, *p =* .001). The region with the highest contribution to the model was the left ventrolateral prefrontal cortex. Results were confirmed in the second sample (*r =* .33, *p =* .01; mean squared error = 8.61, *p =* .01), in which the severity of this symptom was predicted using a bilateral ventrolateral prefrontal cortical mask (*r =* .33, *p =* .009, mean squared error = 9.37, *p =* .04).

**Conclusions:**

The severity of a specific hypo/mania-related symptom was predicted from patterns of whole-brain activation in two independent samples. Given that emerging manic symptoms predispose to bipolar disorders, these findings could provide neural biomarkers to aid early identification of individual-level bipolar disorder risk in young adults.

Bipolar spectrum disorder (BPSD) is a common and debilitating psychiatric disorder, with the 12-month prevalence of BPSD being >2.6% in the United States (1) and the lifetime prevalence being >4.5% 2, 3, 4, 5. Furthermore, BPSD is the fourth leading cause of disability in the world (6). Yet, it remains extremely difficult to identify those young adults who are at risk for development of future BPSD. While having a family history of BPSD in first-degree relatives confers up to a 10-fold higher chance of BPSD (7) depending on the presence or absence of subthreshold BPSD symptoms, presence of such family history does not provide objective biomarkers to guide treatment choice and novel interventions for individuals at risk for BPSD. Thus, it is imperative that objective biomarkers of BPSD risk in young adulthood are identified to enable early and accurate identification of those young adults who are most at risk of developing BPSD, provide biomarkers that distinguish between risk for future BPSD versus risk for the broader range of anxiety and affective disorders, and identify biological targets to ultimately guide treatment choice and novel interventions to delay, or even prevent, development of BPSD in vulnerable young individuals. Furthermore, identifying such biomarkers in young adulthood provides biological targets at a critical period when neurodevelopment is still occurring 8, 9, 10, so that these interventions can take advantage of the plasticity of the brain to minimize development of long-term neural abnormalities.

A way forward in the search for biomarkers of BPSD risk is to identify biomarkers of behaviors that predispose to BPSD. One such behavior is the complex behavioral trait, impulsive sensation seeking (ISS), which comprises the component traits of impulsivity; behavior characterized by little or no forethought, reflection, or consideration of the consequences; and sensation seeking, the tendency and willingness to seek, and take risks for, novel and intense sensations and experiences (11). High levels of ISS are evident in adults with BPSD 12, 13, 14, 15, and various components of ISS also predispose to future BPSD in young adults 16, 17, 18. Specifically, higher scores on several ISS component traits (e.g., impulsivity) are positively associated with (16), and account for 27% of (17), current hypo/mania severity in young adults with subthreshold hypo/manic symptoms. Furthermore, previous studies have shown that subthreshold levels of hypo/mania symptoms confer risk for future BPSD 19, 20. Thus, both higher severity ISS and subthreshold hypo/mania symptoms are associated with risk for future BPSD, likely because of the relationship between higher severity ISS and presence of hypo/mania.

Uncertain reward expectancy (RE) is a reward-striving context involving subjective evaluation of future rewards, in which elevated subjective evaluation of potential future rewards may trigger impulsive thoughts and decisions about these potential rewards in individuals with BPSD and in those with high levels of impulsivity and sensation seeking at risk of these disorders, predisposing to hypo/mania 16, 17. Elucidating the neural basis of subjective evaluation of future rewards during RE in individuals with BPSD is thus a promising way to identify neural biomarkers of BPSD. We previously reported a steeper increase in left uncertain RE-related ventrolateral prefrontal cortical (vlPFC) and ventral striatal activation with greater likelihood (expected value) of rewards in remitted euthymic BPSD versus healthy adults 21, 22, and greater left vlPFC activation to uncertain RE in adults with higher levels of sensation seeking and impulsivity (22).

Most clinical neuroimaging studies use standard statistical approaches to identify group-level patterns of neural activation that distinguish different diagnostic groups. The limitation of these approaches, however, is their inability to make predictions at the individual subject level. Pattern recognition approaches can identify multivariate patterns of brain activation that are predictive of diagnosis or future outcomes 23, 24, 25. It should be emphasized that in the context of pattern recognition, the models’ predictive performance (or predictive power) is measured on “new data” (or new subjects) that were not used to train or fit the model. More specifically, the pattern recognition framework consists of two phases: training and testing. During the training, the model learns a relationship between a set of patterns (e.g., multivariate patterns of brain activation) and labels (e.g., a clinical scores), and during the testing, given new individual patterns (e.g., patterns of brain activation from new subjects), the model predicts their labels. The model performance is then measured by comparing the real and predicted labels for the test data (or test subjects).

One limitation of pattern recognition approaches when applied to whole-brain patterns, however, is that, owing to their multivariate nature, it is difficult to identify the contribution of specific neural regions to the predictive model. As the model (and predictions) is based on the whole multivariate pattern, it is not possible to make local inferences about specific voxels; that is, all voxels with nonzero weights contribute to the model/predictions (26). Indeed, in recent years, there has been a huge debate in the neuroimaging field on how to interpret weight maps of pattern recognition approaches 27, 28, 29. The multiple kernel learning (MKL) approach has been proposed as an approach that can identify a subset of relevant brain regions for the predictive model (29). The MKL models the whole-brain multivariate pattern as a combination of regional patterns and learns the contribution of each neural region for the predictive model. Thus, regions that carry more information about the variable being predicted will have a higher contribution to the model characterized by the region’s weight. Neural regions can then be ranked according to their weights or contributions, which facilitates interpretation of the predictive model in terms of contributions of different neural regions.

In the present study, our first aim was to use MKL regression with functional neuroimaging data to identify individual-level patterns of uncertain RE-related brain activation predictive of the severity of behaviors linked with ISS, and thus predictive of risk factors for future BPSD. By recruiting participants transdiagnostically, we ensured that participants were recruited across a large range of severity on many different behavioral traits and symptoms, including ISS and subthreshold hypo/mania symptoms, and were thus recruited across a range of risk for future BPSD. We also included in our analyses measures of other behaviors and symptoms that are not associated specifically with risk for future BPSD, e.g., anxiety and anhedonia, to determine the specificity of any neuroimaging findings to the determination of ISS severity, as opposed to the severity of other, non-ISS, behaviors. Our previous group-level neuroimaging findings allowed us to hypothesize that the severity of ISS-related behavioral traits and symptoms predisposing to future bipolar spectrum disorders, including impulsivity, sensation seeking, and subthreshold hypo/mania-related symptoms, would be associated with uncertain RE-related activation specifically in reward circuitry regions, including the left vlPFC and ventral striatum. Given the importance of confirming findings in independent samples in clinical neuroscience 30, 31, our second aim was to confirm the findings from the first sample, in an independent sample of transdiagnostically recruited young adults.

## Methods and Materials

### Participants

Neuroimaging data were employed from two independent samples of young adults from the DIAMOND (Dimensions of Affect, Mood, and Neural Circuitry Underlying Distress) study (R01MH100041). The first sample comprised 56 young adults ranging from 18 to 25 years of age (mean age 21.9 ± 2.2 years, 42 women). The second sample (confirmatory sample) comprised 36 young adults ranging from 18 to 25 years of age (mean age 21.2 ± 2.2 years, 24 women). The first sample was included in previous studies using conventional univariate analyses, which examined other brain-behavior relationships 22, 32. The second sample was an independent sample not used in these previous studies. All participants were seeking help for psychological distress, including depressive and anxiety symptoms, and other behavioral and emotional problems such as failing to cope with everyday stressors and interpersonal relationships, irrespective of having a DSM-5 diagnosis or not, defined using the Structured Clinical Interview for DSM-5 (33). The samples had a variety of DSM-5–defined diagnoses, but not BPSD: depressive disorder (*n =* 29), anxiety disorder (*n =* 47), eating disorder (*n =* 3), externalizing disorder (*n =* 13), trauma-related disorder (*n =* 10), sleep disorder (*n =* 19), somatoform disorder (*n =* 3), and adjustment disorder (*n =* 3). Some participants in both samples were below the threshold for any psychiatric disorder (*n =* 22). Only four participants were taking psychotropic medication. No participant stopped medication to participate in the study. See Supplemental Tables S2 and S6 for a complete description.

The study protocol was approved by the University of Pittsburgh Institutional Review Board, and all participants provided informed consent. The full description and exclusion criteria at screening for all participants is in the Supplement.

### Clinical Scales

The pattern regression models included 21 different scales and subscales from the DIAMOND study, which measured a range of ISS-related behavioral traits and other behavioral traits and symptoms, e.g., anxiety and anhedonia, and included 1) the Urgency, Premeditation, Perseverance, Sensation Seeking, Positive Urgency, Impulsive Behavior Scale (34); 2) the Zuckerman Sensation Seeking Scale (11); 3) the Behavioral Activation System (35); 4) the Mood Spectrum Self-Report behavioral trait dimensions (36); 5) the Snaith-Hamilton Pleasure Scale (37); and 6) the Moods and Anxiety Symptom Questionnaire (38). The full description of all scales and subscales is in Supplemental Tables S1 to S4.

### Functional Magnetic Resonance Imaging Paradigm

Details of the experimental design, imaging acquisition, preprocessing steps, and general linear model analysis are described in the Supplement. In summary, we employed a 16-minute event-related card-guessing game from a previous study (22). The trial structure comprised a choice phase, an anticipation phase, numerical feedback, and feedback arrow (win, loss, and neutral). During the choice phase, individuals guessed via button press whether the value of a visually presented card was high or low (4 seconds: presentation of a question mark). In the anticipation phase, an expectancy cue was then presented for 2 to 6 seconds (jittered), with four types of cues/trial types described below. The outcome then appeared for 1 second (the number for 500 ms and then the feedback arrow for 500 ms), followed by a 0.5- to 1.5-second intertrial interval. Individuals practiced the task before the scan. The four trial types were as follows: expectation of possible win, followed by win outcome (win trials) or no change (disappointment trials); expectation of possible loss, followed by loss (loss trials) or no change (relief trials); mixed win/loss trials, followed by win or loss; and neutral trials, followed by no change. The paradigm was administered in two 8-minute blocks, with 48 trials per block: 12 trials each for each trial type and 50% chance of each outcome. Trials were presented in a random order with predetermined outcomes.

Functional magnetic resonance imaging data were preprocessed using a combination of software packages (SPM, FSL, AFNI) implemented in Nipype (39). The regressor of primary interest was RE, a parametric modulator coupled to the duration of the expectancy period, which reflected the expected value of the potential future reward. For each participant, the whole-brain pattern of activation to the RE regressor was used in the pattern regression analyses.

### Pattern Regression Analysis

Pattern regression analyses were implemented in PRoNTo v2.1 (26). MKL pattern regression was applied to predict each of the different clinical scales and subscales from the individual-level whole-brain pattern of activation during RE. The MKL regression approach models the whole brain as a combination of regional patterns and therefore learns the contribution of different brain regions to the predictive model (40). As the MKL model currently implemented in PRoNTo assumes sparsity in the kernel combination [SimpleMKL (41)], it selects only a subset of neural regions to perform the regression; the remaining regions have a null contribution to the model. Regions were defined using an anatomical template [Automated Anatomical Labeling template (42)], which splits the brain into 116 anatomical regions. For each region, a linear kernel was computed based on the regional pattern of activation containing all voxels within the region. The kernels were normalized (to compensate for the fact that the number of voxels varies among brain regions) and mean centered using standard kernel operations implemented in PRoNTo. Age and gender were included as covariates, using a regression model that separates training and testing data, as previously described (43).

**Figure 1:**
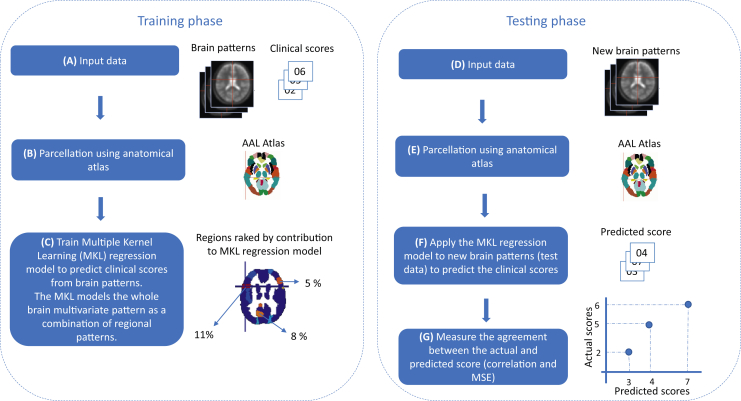
Multiple kernel learning (MKL) framework. Training phase: **(A)** the MKL regression model is trained by providing examples that pair a contrast image from the general linear model (brain patterns) and a clinical score. **(B)** The MKL framework uses a predefined anatomical template to segment the contrast images into 116 anatomical brain regions. **(C)** The MKL simultaneously learns the contribution of each region for the decision function (region weights or contribution) and within each region the contribution of each voxel (voxel weights). Testing phase: **(D)** During the testing phase, a new contrast image (brain patterns) of a test subject is given as input for the MKL model. **(E)** This contrast image is parcellated using anatomical atlas. **(F)** The MKL regression model is applied to the segmented contrast image to predict the clinical score. **(G)** The model performance is evaluated using two metrics to measure the agreement between the predicted and the actual clinical scores: Pearson’s correlation coefficient (*r*) and mean squared error (MSE). AAL, Automated Anatomical Labeling.

A nested threefold cross-validation procedure was used to train the model for each clinical scale and subscale, with the same cross-validation scheme for the internal and external loop. The external loop was used for assessing the model's performance, and the internal loop was used for optimizing model hyperparameters (soft-margin parameter C for the SimpleMKL), using mean squared error (MSE) as the optimization criterion. The performance of the regression models was measured using two metrics of agreement between the predicted and the actual scores: Pearson’s correlation coefficient (*r*) and MSE. Finally, a permutation test was applied to compute the significance of the models, and Bonferroni correction was used to account for multiple comparisons (21 scales), using a significance threshold of .05/21 = .0024 (Figure 1).

In the second sample (confirmatory analysis), the same analysis was performed, but using only the scale that was significantly predicted from patterns of brain activation to RE in the first sample. Here, we tested whether the spatial pattern within the region identified by MKL regression as contributing most to the models in the first sample could predict the scale in the second sample. Thus, we used a bilateral vlPFC mask constructed using a WFU PickAtlas tool available in SPM12 to select the regions for the pattern regression analysis. In addition, we performed exploratory whole-brain MKL predictive models in this second sample.

### Model Interpretation

The MKL model computes two sets of weights, the kernel weights and the voxel weights. The kernel weights represent the contribution of each region (region weights) to the predictive model, and the voxel weights represent the contribution of each voxel within the regions to the predictive model. Both sets of weights can be explicitly computed and plotted as brain images. The kernel or region weights thus enable interpretation of the predictive model in terms of contributions of anatomically defined brain regions.

## Results

### Pattern Regression Analysis in the First Sample

**Figure 2:**
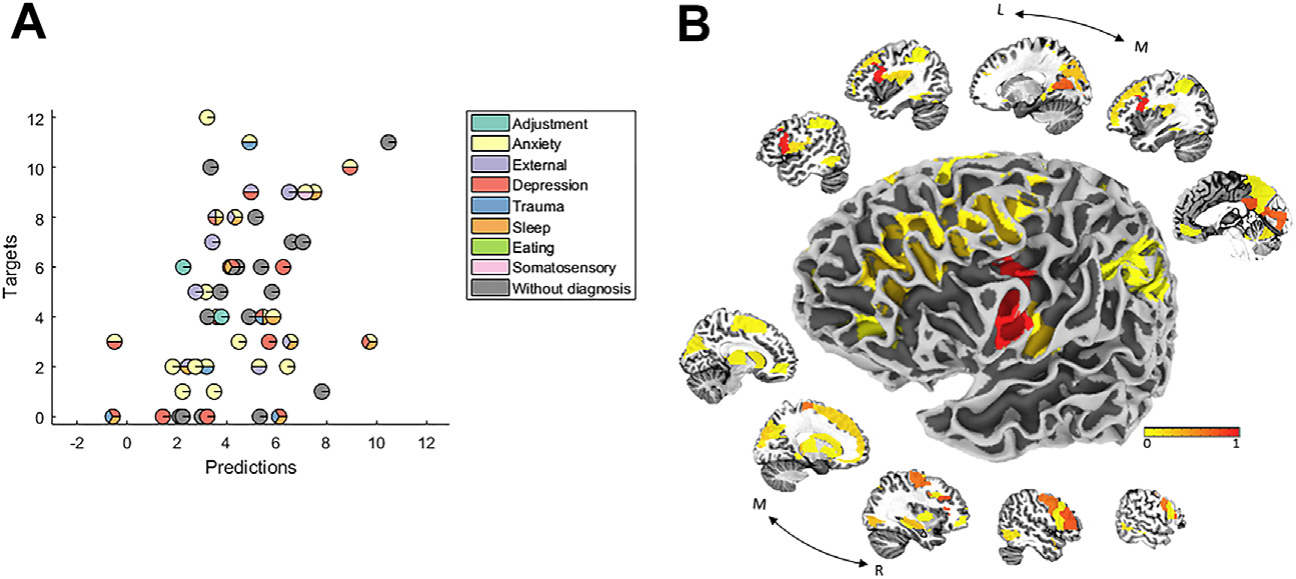
**(A)** Scatter plot between the actual and predicted energy-manic symptom scores for the model based on patterns of whole-brain activation during uncertain reward expectancy using a threefold cross-validation scheme. The correlation coefficient (*r*) and the mean squared error between the actual and predicted energy-manic symptom scores were .42 (*p =* .001) and 9.93 (*p =* .001), respectively. For visualization purposes, subjects were color coded according to the categorically defined diagnoses to stress the transdiagnostic nature of these results. Some subjects, however, presented with symptoms that did not meet the threshold for a DSM-5 diagnosis. **(B)** Weight map showing the contribution of the different brain regions for predicting the energy-manic symptom score from patterns of whole-brain activation during uncertain reward expectancy. The region with the highest contribution according to the multiple kernel learning predictive model was the left ventrolateral prefrontal cortex.

Among all tested scales, after correcting for multiple comparisons, the MKL regression models predicted only the (subthreshold) severity of a hypo/manic symptom, the energy-manic subdomain of the Mood Spectrum Structured Interviews (hereafter referred to as “energy-manic symptom”), a scale measuring lifetime experience of elevated energy associated with hypo/mania (36), from the pattern of whole-brain activation to RE (*r =* .42, *p =* .001; MSE = 9.93, *p =* .001) (Table 1). Figure 2A shows the scatter plot between the predicted and actual energy-manic symptom severity. For visualization purposes, participants were color coded according to their categorically defined diagnosis. This figure emphasizes that the MKL model is able to predict energy-manic symptom cutting across different categorically defined diagnoses. Figure 2B displays the corresponding weights map showing the contribution of the different brain regions to the MKL predictive model. The neural region with highest contribution was the left frontal inferior operculum, part of the left vlPFC. The complete list of neural regions that contributed to the MKL regression model is in Supplemental Table S5.

**Table 1:** Measures of Agreement Between Actual and Predicted Energy-Manic Symptom Severity Based on Patterns of Whole-Brain Activation During Uncertain Reward Expectancy After Controlling for Covariates (Age and Gender) in the First Sample

	*r*	*p* Value	MSE	*p* Value
Value	.42	.001^a^	9.93	.001^a^

MSE, mean squared error.

a Bonferroni correction was used to control for multiple comparisons (21 scales), using a significance threshold of .05/21 = .0024.

### Confirmatory Pattern Regression Analysis in the Second Sample

**Figure 3:**
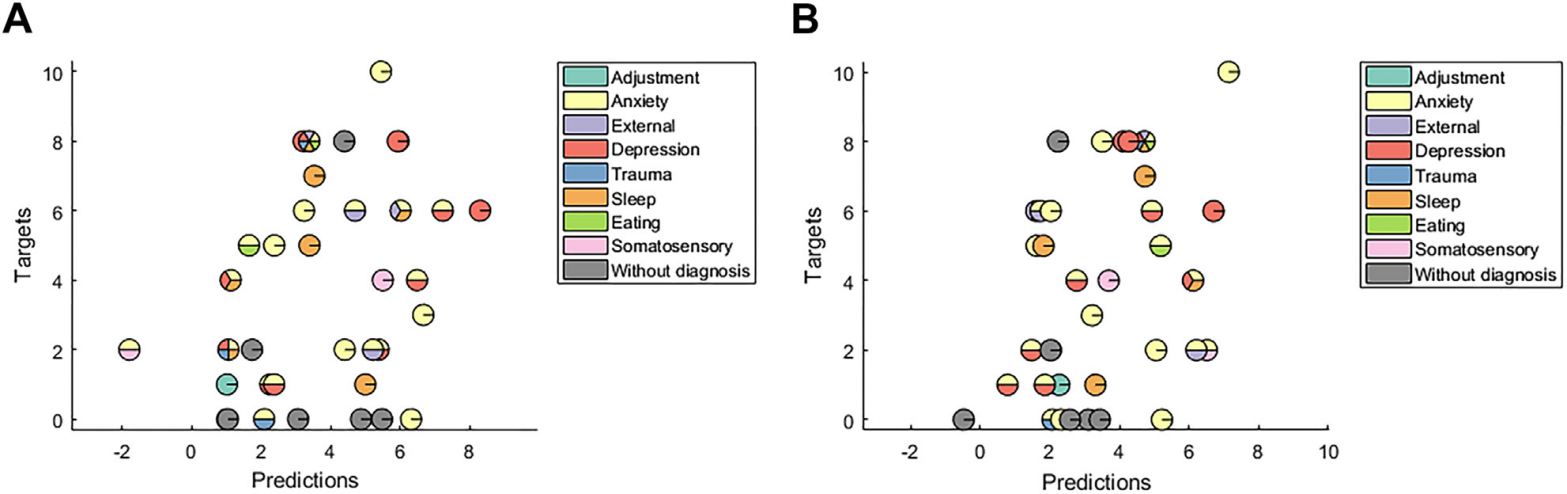
**(A)** Scatter plot between the actual and predicted energy-manic symptom scores for the model based on the pattern of activation within the bilateral ventrolateral prefrontal cortex during uncertain reward expectancy in the second sample. The correlation coefficient (*r*) and the mean squared error between the actual and predicted energy-manic symptom scores were .33 (*p =* .009) and 9.37 (*p =* .04), respectively. **(B)** Scatter plot between the actual and predicted energy-manic symptom scores for the model based on patterns of whole-brain activation during uncertain reward expectancy in the second sample. The correlation coefficient (*r*) and the MSE between the actual and predicted energy manic scores were .33 (*p =* .01) and 8.61 (*p =* .01), respectively. For visualization purposes, subjects were color coded according to the categorically defined diagnoses to emphasize the transdiagnostic nature of the findings. Some subjects, however, presented with symptoms that did not meet the threshold for a DSM-5 diagnosis.

Our main aim with the confirmatory analysis was to examine whether the multivariate pattern within the bilateral vlPFC mask could predict energy-manic symptom severity in the independent second sample. This bilateral vlPFC mask-focused approach significantly predicted the severity of this symptom in this independent sample (*r =* .33, *p =* .009; MSE = 9.37, *p =* .04) (Table 2). The exploratory whole-brain MKL predictive model in this second sample also significantly predicted the severity of this symptom (*r =* .33, *p =* .01; MSE = 8.61, *p =* .01); however, there were some differences among the regions selected in the first and second samples. The complete list of neural regions that contributed to the MKL regression model using the whole brain in the second sample is in Supplemental Table S6. Figures 3A and B show scatter plots between the actual and predicted energy-manic symptom severity for the bilateral vlPFC model, and the whole-brain MKL, respectively, in the second sample. For visualization purposes, participants were color coded according to their categorically defined diagnosis to emphasize the transdiagnostic nature of these results. As noted above, this figure illustrates that the MKL models are able to predict energy-manic symptom severity in participants across different categorically defined diagnoses.

**Table 2:** Measures of Agreement Between Actual and Predicted Energy-Manic Symptom Severity Based on Patterns of Bilateral vlPFC Activation and Whole-Brain Activation During Uncertain Reward Expectancy After Controlling for Covariates (Age and Gender) in the Second Sample

	*r*	*p* Value	MSE	*p* Value
vlPFC	.33	.009	9.37	.04
Whole Brain	.33	.01	8.61	.01

MSE, mean squared error; vlPFC, ventrolateral prefrontal cortex.

## Discussion

In the present study, we demonstrate that the severity of a specific symptom, heightened energy associated with hypo/mania, predisposing to future risk for BPSD, can be predicted from patterns of neural activation during uncertain RE in a sample of transdiagnostically recruited young adults experiencing psychological distress, but who have not yet developed BPSD. Furthermore, the importance of the vlPFC, which was identified as the neural region contributing most to the MKL regression model in the first sample, was confirmed in a second independent sample of transdiagnostically recruited young adults. These findings show for the first time that individual-level patterns of whole-brain activation, by predicting the severity of a symptom associated with risk for BPSD in on-BPSD young adults, are potential neural biomarkers that may be used in future studies to identify those individuals most at risk of developing these disorders.

A major finding was the importance of RE-related left vlPFC activation to the MKL regression model in the first sample. Our previous findings highlight the role of this region as a potential neural biomarker of BPSD in individuals with, and those at risk for, this disorder 21, 22, 44. The left vlPFC links stimuli to specific reward outcomes 45, 46 and is implicated in concrete decision making focusing on immediate rewards 47, 48. The left laterality of the vlPFC likely reflects the left frontal cortex’s role in approach behaviors (49). Thus, abnormally elevated RE-related left vlPFC activation in individuals with bipolar disorder may reflect greater positive subjective evaluation of the probability for immediate future rewards and predispose to hypo/mania. Our findings in the second sample (and the fact that the regions with correlated information/patterns might not be selected by the used MKL approach) suggest, however, that we cannot exclude the fact that the right vlPFC may also have predictive information about energy-manic symptom severity. Future work should determine if the lateralization of the vlPFC previously observed in univariate statistical analyses (22) is also observed in multivariate pattern recognition analyses. Overall, our present findings demonstrate for the first time that RE-related patterns of activation in the vlPFC play an important role in predicting individual-level severity of a specific ISS-related symptom, energy-manic symptom severity, reflecting heightened hypo/mania-related energy. Moreover, previous studies suggest that the energy-manic symptom is a key subdomain of the Mood Spectrum Self-Report discriminating individuals with BPSD from those with major depressive disorder 50, 51, 52. Together, these findings suggest the potentially important role of RE-related vlPFC activation in predicting severity on a hypo/manic symptom associated with BPSD risk in young adults. Future longitudinal studies should be performed to confirm this hypothesis.

It should be noted that energy-manic scores of participants in the present study were lower than the range previously observed in adults with BPSD and closer to scores in healthy participants (see Supplement), as described in several studies 36, 51, 53, 54. This is expected, as none of the participants had a diagnosis of BPSD. However, the range of energy-manic scores in participants in the present study indicates the inclusion of participants with higher scores that are in the BPSD range, highlighting the heterogeneity of the participant population regarding diagnosis and BPSD risk, and in accordance with the Research Domain Criteria recommendation of examining populations along a continuum from normal to pathological to identify biomarkers reflecting pathophysiological processes 55, 56.

One important methodological innovation of this study was the use of MKL regression to identify the specific contribution of different neural regions to the predictive model. Another important aspect of the present study is the confirmation of the main results (importance of the vlPFC activation in predicting energy-manic symptom) in a second independent sample. As highlighted previously 30, 31, of 49 clinical studies with more than 1000 citations, findings from only 44% of these studies were replicated. Lack of reproducibility is a key problem for science in general, especially for medical sciences, in which robust and reproducible findings have potential to lead to improvement in diagnosis and treatment development. Finally, the transdiagnostic approach of the present study, in accordance with the National Institutes of Health Research Domain Criteria dimensional approach to the study of psychiatric disorders 55, 56, ensured that our findings were generalizable across different disorders rather than being specific to a given disorder.

There were some limitations to the present study. The main limitation was the sample size of the second sample. Although we were able to confirm that vlPFC patterns of activation (regions identified as the most relevant for the predictive model in the first sample) were predictive of energy-manic symptoms in the second, independent, sample, there were some differences in the results obtained for both samples. In particular, the vlPFC was not selected by the exploratory whole-brain MKL model in the second sample as the most relevant for the predictions. One potential explanation for the difference in the regions selected between the two samples is the fact that the MKL implementation used in the present study, simple MKL (41), is based on an L1-norm regularization, which, like LASSO regression (57), might not select regions with correlated information; that is, if two regions have correlated information and are both relevant for the predictions, only one of them will be included in the model. Another potential justification is the fact that the second sample was much smaller than the first one and therefore might produce unstable results in terms of regions selected. An additional limitation is that even though the MKL approach is able to identify a subset of important regions for the predictive model, it does not answer the question of why these regions are individually relevant. Alternative analyses (such as the mass-univariate general linear model) are needed to examine the association between the signal in individual regions and energy-manic symptom severity. Future studies should aim to replicate findings in larger samples and in other BPSD at-risk populations, including individuals with genetic predisposition to developing this disorder.

In summary, in the present study, individual-level severity of a specific hypo/mania symptom related to risk for BPSD, the energy-manic subdomain of the Mood Spectrum Self-Report, was predicted from patterns of uncertain RE-related whole-brain activation in two independent samples of transdiagnostically recruited young adults, with the left vlPFC identified as the neural region contributing most to the predictive model in the first sample. Our previous findings indicate a positive association between the magnitude of left vlPFC activation to uncertain RE and the magnitude of ISS in young adults (22), as well as significantly greater activation in this region during this context in individuals with established BPSD relative to healthy individuals (21). The present machine learning study now provides further evidence that the vlPFC multivariate pattern of activation during uncertain RE is significantly associated with the severity of a hypo/mania symptom that, in turn, is associated with greater future BPSD risk, heightened energy (measured by the energy-manic subdomain of the Mood Spectrum Structured Interviews). Thus, the present study builds on present research findings and indicates that the vlPFC pattern of activation may indeed be an objective neural marker of a hypo/manic symptom that denotes future risk for BPSD at the individual-person level. These findings can aid early identification of BPSD risk in young adults and provide neural targets to guide the development and choice of early therapeutic interventions, potentially reducing the significant social costs and deleterious outcomes associated with the disorder (58), in these vulnerable individuals.
