# Supplementary material for: Predicting Bipolar Disorder Risk Factors in Distressed Young Adults From Patterns of Brain Activation to Reward: A Machine Learning Approach
Source: Biol Psychiatry Cogn Neurosci Neuroimaging. 2019 Aug;4(8):726–33. doi: 10.1016/j.bpsc.2019.04.005 (PMC6682607; doi:10.1016/j.bpsc.2019.04.005)
Supplement: Supplemental Material [file mmc1.pdf]

Predicting Bipolar Disorder Risk Factors in Distressed Young Adults From Patterns of Brain Activation to Reward: A Machine Learning Approach

Supplementary Information

Supplementary Methods

Exclusion Criteria at Screening for All Participants

1. History of head injury, neurological, pervasive developmental disorder (e.g. autism), or systemic medical disease (that could impact fMRI scans; from medical records and report by each potential participant); 2. Mini-Mental State Examination (1) (cognitive state) score<24; 3. premorbid NAART IQ (2) estimate<85; 4. visual disturbance (<20/40 Snellen visual acuity); 5. left or mixed handedness (3), to ensure a uniform hemispheric dominance for interpretation of neuroimaging data; 6. Alcohol/substance use disorder (including nicotine) and/or illicit substance use (except cannabis) over the last 3 months, determined by the Structured Clinical Interview for DSM5 (SCID-5) (4) (and psychiatric records, if available). Lifetime/present cannabis use (non substance use disorder levels) was allowed, given its common usage in 18-25 year-olds (5). Urine tests on the scanning day excluded individuals with current illicit substance use (except cannabis); salivary alcohol tests excluded individuals who were intoxicated on the scanning day. Alcohol/nicotine/caffeine/cannabis use (below SCID-5-defined substance use disorder levels) per week was noted; 7. MRI exclusion criteria, including metallic foreign objects, such as aneurysm clips or pacemakers, or a questionable history of metallic fragments, proneness to panicking in enclosed spaces, and a positive pregnancy test for female individuals or self-reporting of pregnancy; 8. being treated with any psychotropic medication for >2 weeks, given the potentially confounding effect of such medication on neuroimaging measures in the proposed study.

Supplementary Table S1. Scales and sub-scales tested during exploratory analysis

Clinical Scales	Subscales	Sample 1	
		Range	Mean (SD)
**UPPS-P Impulsive Behavior Scale**		1.33- 3.38	2.26 (0.4)
**Sensation Seeking Scale**	Boredom Susceptibility	0 - 8	3.18 (1.6)
	Disinhibition	0- 10	4.14 (2.6)
	Experience Seeking	1 - 10	5.20 (2.0)
	Thrill and Adventure Seeking	0-10	5.36 (3.0)
**Behavioral Activation System**	Fun Seeking Activation	6-16	12.02 (2.6)
	Reward Responsiveness Activation	12-20	17.12 (2.0)
	Total Score	23-51	40.52 (6.3)
**MOODS-self report (SR)**	Mood Manic	1-26	11.36 (6.2)
	Cognition Manic	0-22	7.57 (4.7)
	Energy Manic	0-12	4.68 (3.4)
	Mood Total Score	26-142	73.98 (23.3)
**Snaith Hamilton Pleasure Scale (SHAPS)**	Total Score	14-42	27.12 (7.4)
**Moods and Anxiety Symptom Questionnaire (MASQ)**	MASQ90_AD	39-101	74.80 (15.4)
	MASQ90_AA	17-65	29.20 (11.5)
**State-Trait Anxiety Inventory (STAI)**	STATE Anxiety Total Score	20-75	48.23 (11.4)
	TRAIT Anxiety Total Score	25-75	56.26 (10.8)
**Hamilton Depression Rating Scale**	Total Score	4-28	15.53 (6.6)
**Young Mania Rating Scale**	Total Score	0-19	3.57 (3.1)
**NEO Personality Inventory**	Neuroticism	51-170	117.88 (20.5)
	Extraversion	26-170	99.78 (28.2)

*For sample 2 (confirmatory analysis) only the energy-manic subscale was tested (mean=3.69 (SD: 3.0); range 0-10). SD – Standard deviation.

Clinical Scales

UPPS-P Impulsive Behavior Scale (6). The UPPS-P Impulsive Behavior Scale captures the multidimensional nature of impulsivity. It was derived by including existing scales of impulsivity in factor analysis. Participants respond to each item using a 4-point Likert scale: 1 (Agree Strongly), 2 (Agree Some), 3 (Disagree Some), and 4 (Disagree Strongly). There are four subscales: Negative Urgency, the tendency to act rashly in intense negative emotional states; Lack of Premeditation, the tendency to act without forethought and planning; Lack of

Perseverance, the tendency not to finish tasks and Sensation Seeking, the tendency to seek sensory pleasure and excitement.

The Zuckerman Sensation Seeking Scale (7). The Zuckerman Sensation-Seeking Scale-V (SSS) consists of 40 forced-choice questions designed to assess individual differences in optimal level of stimulation. The Zuckerman Sensation Seeking Scale includes standardized measures of sensation-seeking subcomponents: experience seeking; thrill seeking; disinhibition; and boredom susceptibility. Here, SSS-V was scored as a general measure of sensation-seeking by summing all 4 factors.

The Behavioral Activation System scale (BAS5) (8). The Behavioral Activation System scale (BAS) reflects reward sensitivity and sensation-seeking subcomponents: drive; fun seeking; and reward responsivity. The BAS consists of 13 items with scores between 1-4 (range=13-52).

MOODS-self report (SR) (9). The Mood Spectrum Structured Interviews were developed to assess, in addition to typical DSM core symptoms of Bipolar Disorder, dimensions of subthreshold features of emotional regulation, as well as trait-like behavioral features that arise as a result of coping with psychopathology related to Bipolar Disorder (9,10). The SCI-MOODS was the first version of the questionnaire and the MOODS-SR is its self-report version. The instruments consist of 161 items coded as present or absent for one or more periods of at least 3-5 d through the subject's lifetime or over the past week or month. The MOODS-SR was originally composed of four domains: 1) the Neurovegetative domain, assessing disturbances and rhythmic changes in feelings, eating attitudes, sexual activity and sleep,

including rhythmic variations in affective and sub-affective symptoms; 2) the Energy levels domain, assessing changes in everyday activities, with special attention devoted to work, hobbies and social life; 3) the Mood domain, exploring the whole realm of depressive and manic symptoms, signs and sub-threshold manifestations of mood dysregulations; and 4) the Cognitive domain, assessing changes in the cognitive realm that occur together with mood dysregulation (9).

Snaith Hamilton Pleasure Scale (SHAPS) (11). This measure was developed to evaluate the level of anhedonia. Subjects are asked to answer 14 questions about hedonic capacity, with scores between 1 and 4. High scores indicate more severe anhedonia.

Moods and Anxiety Symptom Questionnaire (MASQ) anhedonia (AD) subscale (MASQ-AD) (12). The MASQ anhedonia subscale consists of 22 items ranging from 22 to 110. This questionnaire measures symptoms of anhedonia in depression. The participants responded to statement such as “felt like nothing was very enjoyable.” and “felt really slowed down.” This scale has been shown to reflect depressive mood (13) and to predict current and lifetime depressive disorders (14).

Hamilton Depression Rating Scale (HDRS). The Hamilton Depression Rating Scale (15) measures the severity of depressive symptoms. Although the HDRS form consists of 21 items, the scoring is based on the first 17 items.

The Spielberger State-Trait Anxiety Inventory (STAI) (16) scale consists of a four-point Likert scale to assess anxiety. This scale is divided into two sections: trait (STAI-T) and state scale

(STAI-S), each with 20 questions. Trait anxiety is a self-report questionnaire that consists of 20 items ranging from 20 to 80 that represents a general tendency an individual has to respond with anxiety in response to environmental stimuli. State anxiety, also consists of 20 items ranging from 20 to 80, captures a transient condition characterized by tension, apprehension, and hyperactivity of the autonomic nervous system.

Young Mania Rating Scale. The Young Mania Rating Scale (YMRS) (17) is one of the most frequently utilized rating scales to assess manic symptoms at baseline and over time. The scale is generally done by a clinician or other trained rater with expertise with manic patients. The scale has 11 items with each item given a severity rating. There are four items that are graded on a 0 to 8 scale (irritability, speech, thought content, and disruptive/aggressive behavior), while the remaining seven items are graded on a 0 to 4 scale.

NEO Personality Inventory (18) is a 60-item personality questionnaire consisting of 5 sub-scales: Neuroticism (N), extraversion (E), openness (O), agreeableness (A), and conscientiousness (C). This instrument is based on the idea that identification of basic dimensions of human personality is possible via the application of factor analytic techniques to verbal descriptors of human traits.

Image Acquisition

Neuroimaging data were collected using a 3.0 Siemens Trio MRI scanner (First Sample= 56 young adults) and Tesla Prisma scanner (Second Sample (confirmatory analyses) = 36 young adults) at the MRI Research Center at the Presbyterian Hospital, Pittsburgh. For the Siemens Trio MRI scanner, Blood-oxygenation-level-dependent (BOLD) images were acquired with a

multi-band gradient echo EPI sequence (18x(MB)3=54 slices; 2.3mm isotropic voxels; TR/TE=1500/30msec; Field of View=220x220mm; matrix 96x96; Flip Angle 55°, Bandwidth 1860 Hz/Px). Structural 3D axial MPRAGE images were acquired in the same session (TE: 3.19ms; TR:1500ms; Flip Angle8°; FOV=256x256mm; 1mm isotropic voxels; 176 continuous slices). For the Prisma scanner, the structural data were acquired with following parameters 3D axial MPRAGE images (TR=1520ms, TE=3.17ms, Flip Angle 8°, FOV=256×256mm, $1mm^3$ isotropic voxels, 176 continuous slices; 4 min, 50s). BOLD functional images_were acquired with a gradient echo echo-planar imaging (EPI); multiband (MB)3 sequence, covering 18x(MB)3 (=54) oblique slices (TR/TE=1500/30ms; $2.3mm^3$ slices; Flip angle=55º; FOV=220x220; Matrix=96x96).

fMRI Paradigm: Reward Paradigm Design

We employed a 16-min event-related card-guessing game from previous study (19). Trial structure comprising choice phase, anticipation phase, numerical feedback and feedback arrow (win, loss and neutral). During choice phase, individuals guessed via button press whether the value of a visually-presented card was high or low (4 seconds: presentation of a question mark). In the anticipation phase, an expectancy cue was then presented for 2–6 seconds (jittered), with four types of cues/trial types described below. The outcome then appeared for 1 second (the number for 500ms and then the feedback arrow for 500ms), followed by a 0.5–1.5 second inter-trial interval. Individuals practiced the task before the scan. The four trial types were as follows: expectation of possible win, followed by win outcome (win trials) or no change (disappointment trials); expectation of possible loss, followed by loss (loss trials) or no change (relief trials); mixed win/loss trials, followed by win or loss; neutral trials, followed by no change. The paradigm was administered in 2, 8-minute

blocks, with 48 trials per block: 12 trials each for each trial type; and 50% chance of each outcome. Trials were presented in a random order with predetermined outcomes. Individuals were told that their performance would determine a monetary reward after the scan: $1 for each win and 75 cents deducted for each loss. Total possible earnings were $6.

Data Pre-processing and General Linear Model Analysis

Data were preprocessed using a combination of software packages (SPM, FSL, AFNI) implemented in Nipype (20). Data for each participant were realigned to the first volume in the time series to correct for head motion. Realigned BOLD images were then co-registered with the subject's anatomical image. Distortion was of this image was corrected with a fieldmap, employing the FSL FUGUE package (for 6 participants we missed this information). The anatomical image was normalized to the MNI/ICBM 152 template using a non-linear transformation and segmented into separate tissue types. BOLD images were then transformed to the same space via the segmented structural image (the DARTEL method), at a resolution of 2 mm^3 isotropic voxel size. BOLD images were corrected for activity spikes using the AFNI 3dDespike tool, normalized for intensity and then spatially smoothed with a FWHM of 6 mm, using FSL's SUSAN adaptive smoothing method.

A first-level fixed effect general linear model (GLM) was constructed for each participant using Statistical Parametric Mapping software, Version-8 (SPM8). The regressor of primary interest was RE (reward expectancy), a parametric modulator coupled to the 2–6 s duration expectancy period, which reflected the expected value of the arrow. It was set to +0.5 for the possible win condition (50% chance of winning $1), –0.375 for the possible loss condition (50% chance of losing $0.75), +0.125 for the mixed condition (50% chance of winning $1; 50%

chance of losing $0.75), and zero for the neutral condition. Other regressors reflecting prediction error and the range of outcome values were also included in the GLM, as were omission errors and regressors coupled to the question mark, the card and the outcome (19). To create the GLM from the design, the canonical hemodynamic response function was convolved with each regressor. Movement parameters from the realignment stage were entered as covariates of no interest to control for participant movement. A regressor to correct for physiological fluctuations was included, derived from the mean signal within white matter, cerebrospinal fluid and high temporal standard deviation voxels (21,22). A high-pass filter (60 s), and autoregressive (AR(1)) modelling were also implemented during first-level model fitting. For each participant, wholebrain activation to the RE regressor was used for the pattern regression analysis.

Finally, a customized mask was created to include only brain voxels, which were common to all participants in the contrast imaging (i.e. we excluded voxels which had a NaN (Not a Number) in the contrast imaging for at least one participant). We previously noted that this criterion for creating the mask can significantly improve the performance of pattern recognition analyses by decreasing the number of noninformative features/voxels in the model (23).

Supplementary Table S2. Diagnostic distribution separately for sample 1 and sample 2

Present Diagnosis		
	Sample 1	Sample 2
Anxiety	45	61
Depression	32	30
Sleep	21	20
Eating	2	5
Trauma	13	8
External	16	11
Somatosensory	2	5
Adjustment	4	3

Data presented in percentage.

Supplementary Table S3. Diagnostic distribution separately for both testing and training sets in sample 1

	1		2		3	
	Training	Test	Training	Test	Training	Test
Anxiety	48	39	42	50	44	45
Depression	29	39	34	28	33	30
Sleep	26	11	21	22	17	30
Eating	0	6	3	0	3	0
Trauma	16	6	8	22	14	10
External	13	22	19	11	17	15
Somatosensory	2.5	0	2.5	0	0	5
Adjustment	3	6	5	0	3	5

Data presented in percentage.

Supplementary Table S4. Diagnostic distribution separately for both testing and training sets in sample 2

	1		2		3	
	Training	Test	Training	Test	Training	Test
Anxiety	54	75	67	50	63	58
Depression	29	33	29	33	33	25
Sleep	21	17	17	25	21	17
Eating	8	0	4	8	4	8
Trauma	8	8	4	17	13	0
External	17	0	8	17	8	17
Somatosensory	4	8	4	8	8	0
Adjustment	0	8	4	0	4	0

Data presented in percentage.

Supplementary Table S5. Mean and standard deviation of ages and energy-manic scores for each set in sample 1 and sample 2

	Sample 1					
	1		2		3	
	Training	Test	Training	Test	Training	Test
Age	22.1 (2.1)	25.1 (2.3)	23.5 (2.1)	22.4 (2.3)	23.75 (2.3)	21.8 (1.9)
energy/manic scores	4.3 (3.5)	5.0 (3.4)	4.25 (3.4)	5.6 (3.0)	5.3 (3.2)	3.5 (3.5)
	Sample 2					
	1		2		3	
	Training	Test	Training	Test	Training	Test
Age	21.3 (2.3)	21.1 (2.1)	21.2 (2.2)	21.2 (2.2)	21.2 (2.2)	21.3 (2.3)
energy/manic scores	4.1 (3.0)	3.0 (3.1)	3.4 (2.9)	4.2 (3.3)	3.6 (3.2)	3.8 (2.6)

Supplementary Table S6. Lifetime diagnostic distribution separately for sample 1 and sample 2

Lifetime disorders	Sample 1	Sample 2
Mood	75	83
Bipolar	4	0
Unipolar depressive	71	83
Anxiety	57	69
Eating	2	11
Externalizing	25	19
Trauma	18	14
Adjustment	4	3

Data presented in percentage.

Supplementary Results

Supplementary Table S7. The complete list of brain regions that contributed to the MKL regression model for sample 1 (ROI weight > 0%)

ROI Label	ROI weight (%)
Frontal_Inf_Oper_L	12.00
Frontal_Inf_Tri_R	9.52
Cingulum_Post_L	9.28
Calcarine_L	9.03
Precentral_R	7.93
Occipital_Inf_R	4.04
Olfactory_R	4.04
Hippocampus_R	3.89
Occipital_Sup_L	3.72
Temporal_Pole_Mid_R	3.54
Occipital_Sup_R	3.30
Frontal_Sup_R	2.71
Rolandic_Oper_L	2.33
Frontal_Mid_L	2.24
Amygdala_L	2.06
Cerebelum_Crus1_L	1.90
Frontal_Sup_Orb_R	1.89
ParaHippocampal_L	1.86
Temporal_Inf_R	1.63
Vermis_6	1.45
Caudate_R	1.29
Cuneus_R	1.23
Vermis_10	1.02
Pallidum_R	0.98
Temporal_Inf_L	0.97
Supp_Motor_Area_R	0.93
Heschl_L	0.90
Thalamus_R	0.83
Frontal_Inf_Oper_R	0.64
Temporal_Pole_Mid_L	0.57
Precuneus_L	0.56
ParaHippocampal_R	0.36
Vermis_1_2	0.35
Putamen_R	0.27
Parietal_Inf_L	0.22
Frontal_Mid_Orb_R	0.15
Frontal_Mid_Orb_L	0.13
Frontal_Mid_Orb_R	0.08
Frontal_Mid_Orb_L	0.07
Amygdala_R	0.04
Rectus_L	0.01

Supplementary Table S8. The complete list of brain regions that contributed to the MKL regression model for sample 2 (ROI weight > 0%)

ROI Label	ROI weight (%)
Vermis_1_2	16.69
Paracentral_Lobule_R	9.96
Cerebelum_3_L	9.86
Thalamus_L	9.81
Lingual_L	6.40
Cerebelum_8_L	4.97
Heschl_L	4.88
Cerebelum_9_L	4.60
ParaHippocampal_L	4.11
Supp_Motor_Area_L	3.70
Rectus_L	3.01
Temporal_Pole_Mid_R	2.82
Frontal_Mid_Orb_L	2.67
Vermis_8	2.53
Calcarine_R	2.19
Frontal_Sup_R	2.14
Angular_R	1.74
Frontal_Mid_Orb_L	1.60
Cerebelum_3_R	1.56
Cingulum_Post_R	1.31
Parietal_Inf_R	1.13
Olfactory_L	0.90
Amygdala_L	0.60
Vermis_3	0.38
Vermis_10	0.34
Frontal_Inf_Orb_L	0.10

Supplementary References

1. Folstein MF, Folstein SE, McHugh PR. "Mini-mental state". A practical method for grading the cognitive state of patients for the clinician. J Psychiatr Res 1975; 12(3): 189-198.
2. Blair JR, Spreen O. Predicting premorbid IQ: A revision of the national adult reading test. Clinical Neuropsychologist 1989; 3(2): 129-136.
3. Annett M. A classification of hand preference by association analysis. Br J Psychol 1970; 61(3): 303-321.
4. First MB, Williams JBW, Karg RS, Spitzer RL. Structured Clinical Interview for DSM-5—Research Version (SCID-5 for DSM-5, Research Version; SCID-5-RV). . American Psychiatric Association: Arlington, VA., 2015.
5. SAMHSA. 2010 National Survey on Drug Use and Health. Dept. of Health and Human Services, Substance Abuse and Mental Health Services Administration, Office of Applied Studies Rockville, MD, 2011.Whiteside, S. P., & Lynam, D. R. (2001). The five factor model and impulsivity: Using a structural model of personality to understand impulsivity. Personality and Individual Differences, 30(4), 669–689. doi:10.1016/S0191-8869(00)00064-7
6. Zuckerman M. The sensation seeking scale V (SSS-V): Still reliable and valid. Pers Indiv Differ 2007; 43: 1303–1305.
7. Carver CS, White TL. Behavioral inhibition, behavioral activation, and affective responses to impending reward and punishment: the BIS/BAS scales. J Pers Soc Psychol 1994; 67: 319–333.
8. Dell'Osso L1, Armani A, Rucci P, Frank E, Fagiolini A, Corretti G, Shear MK, Grochocinski VJ, Maser JD, Endicott J, Cassano GB. Measuring mood spectrum: comparison of interview (SCI-MOODS) and self-report (MOODS-SR) instruments. Compr Psychiatry. 2002 Jan-Feb;43(1):69-73.
9. Fagiolini A, Dell'Osso L, Pini S, Armani A, Bouanani S, Rucci P, Cassano GB, Endicott J, Maser JD, Shear MK, et al. Validity and reliability of a new instrument for assessing mood symptomatology: the Structured Clinical Interview for Mood Spectrum (SCI MOODS) Int J Meth Psych Res. 1999;8:71-81.
10. Snaith R, Hamilton M, Morley S, Humayan A, Hargreaves D, Trigwell P. A scale for the assessment of hedonic tone the Snaith-Hamilton Pleasure Scale. The British Journal of Psychiatry. 1995;167(1):99–103. doi: 10.1192/bjp.167.1.99
11. Clark LA and Watson D. (1991) Tripartite model of anxiety and depression: psychometric evidence and taxonomic implications. J Abnorm Psychol; 100(3):316-36. PMID:1918611 DOI: 10.1037/0021-843X.100.3.316
12. Nitschke JB, Heller W, Imig JC, McDonald RP, Miller GA. Distinguishing dimensions of anxiety and depression. Cognitive Therapy and Research 2001;25:1–22
13. Bredemeier K, Spielberg JM, Silton RL, Berenbaum H, Heller W, Miller GA.(2010) Screening for depressive disorders using the Mood and Anxiety Symptoms Questionnaire Anhedonic Depression Scale: a receiver-operating characteristic analysis. Psychol Assess 22(3):702-10. doi: 10.1037/a0019915.

14. Hamilton M. (1960) A rating scale for depression. J Neurol Neurosurg Psychiatry; 23:56–62. PMID:14399272 DOI:10.1136/jnnp.23.1.56

15. Spielberger CD, Gorsuch RL, Lushene R. (1983) State-Trait Anxiety Invenstory Test Manual Form Y. Consulting Psychological Press: Palo Alto, CA.

16. Young RC, Biggs JT, Ziegler VE, Meyer DA. A rating scale for mania: reliability, validity and sensitivity. Br J Psychiatry. 1978;133:429–435.

17. Costa PT, McCrae RR (1992) Revised NEO personality inventory (NEO PI-R) and NEO five-factor inventory (NEO-FFI) professional manual. Psychological Assessment Resources Inc., Odessa

18. Chase HW, Fournier JC, Bertocci MA, Greenberg T, Aslam H, Stiffler R, Lockovich J, Graur S, Bebko G, Forbes EE, Phillips ML. A pathway linking reward circuitry, impulsive sensation-seeking and risky decision-making in young adults: identifying neural markers for new interventions. Transl Psychiatry. 2017 Apr 18;7(4):e1096. doi: 10.1038/tp.2017.60.

19. Gorgolewski K, Burns CD, Madison C, Clark D, Halchenko YO, Waskom ML et al. Nipype: a flexible, lightweight and extensible neuroimaging data processing framework in python. Front Neuroinform 2011; 5: 13

20. Behzadi Y, Restom K, Liau J, Liu TT. A component based noise correction method (CompCor) for BOLD and perfusion based fMRI. Neuroimage 2007; 37: 90–101.

21. Fournier JC, Chase HW, Almeida J, Phillips ML. Model specification and the reliability of fmri results: implications for longitudinal neuroimaging studies in psychiatry. PLoS One 2014; 9: e105169.

22. Portugal LC, Rosa MJ, Rao A, Bebko G, Bertocci MA, Hinze AK, Bonar L, Almeida JR, Perlman SB, Versace A, Schirda C, Travis M, Gill MK, Demeter C, Diwadkar VA, Ciuffetelli G, Rodriguez E, Forbes EE, Sunshine JL, Holland SK, Kowatch RA, Birmaher B, Axelson D, Horwitz SM, Arnold EL, Fristad MA, Youngstrom EA, Findling RL, Pereira M, Oliveira L, Phillips ML, Mourao-Miranda J. (2016) Can Emotional and Behavioral Dysregulation in Youth Be Decoded from Functional Neuroimaging? PLoS One; 5;11(1):e0117603. PMID:26731403 DOI: 10.1371/journal.pone.0117603
